# Characteristics of relief and residual low back pain after discectomy in patients with lumbar disc herniation: analysis using a detailed visual analog scale

**DOI:** 10.1186/s12891-021-04015-z

**Published:** 2021-02-11

**Authors:** Hiroshi Takahashi, Yasuchika Aoki, Masahiro Inoue, Junya Saito, Arata Nakajima, Masato Sonobe, Yorikazu Akatsu, Keita Koyama, Yasuhiro Shiga, Kazuhide Inage, Yawara Eguchi, Sumihisa Orita, Satoshi Maki, Takeo Furuya, Tsutomu Akazawa, Tetsuya Abe, Toru Funayama, Hiroshi Noguchi, Kousei Miura, Kentaro Mataki, Yosuke Shibao, Fumihiko Eto, Mamoru Kono, Masao Koda, Masashi Yamazaki, Seiji Ohtori, Koichi Nakagawa

**Affiliations:** 1 Department of Orthopaedic Surgery, Faculty of Medicine, University of Tsukuba, 1-1-1, Tennodai, Tsukuba City, Ibaraki, 305-8575 Japan; 2 Department of Orthopaedic Surgery, Toho University Sakura Medical Center, Sakura, Japan; 3 Department of Orthopaedic Surgery, Eastern Chiba Medical Center, Togane, Japan; 4 Department of Orthopaedic Surgery, Chiba University Graduate School of Medicine, Chiba, Japan; 5 Department of Orthopaedic Surgery, St. Marianna University School of Medicine, Kawasaki, Japan

**Keywords:** Lumbar disc herniation, Residual low back pain, Visual analog scale, Radicular low back pain

## Abstract

**Background:**

Several authors have reported favorable results in low back pain (LBP) for patients with lumbar disc herniation (LDH) treated with discectomy. However, detailed changes over time in the characteristics and location of LBP before and after discectomy for LDH remain unclear. To clarify these points, we conducted an observational study to determine the detailed characteristics and location of LBP before and after discectomy for LDH, using a detailed visual analog scale (VAS) bilaterally.

**Methods:**

We included 65 patients with LDH treated by discectomy in this study. A detailed VAS for LBP was administered with the patient under 3 different conditions: in motion, standing, and sitting. Bilateral VAS was also administered (affected versus opposite side) for LBP, lower extremity pain (LEP), and lower extremity numbness (LEN). The Oswestry Disability Index (ODI) was used to quantify clinical status. Changes over time in these VAS and ODI were investigated. Pfirrmann grading and Modic change as seen by magnetic resonance imaging (MRI) were reviewed before and 1 year after discectomy to determine disc and endplate condition.

**Results:**

Before surgery, LBP on the affected side while the patients were in motion was significantly higher than LBP while they were sitting (*p* = 0.025). This increased LBP on the affected side in motion was improved significantly after discectomy (*p* < 0.001). By contrast, the residual LBP while sitting at 1 year after surgery was significantly higher than the LBP while they were in motion or standing (*p* = 0.015). At 1 year following discectomy, residual LBP while sitting was significantly greater in cases showing changes in Pfirrmann grade (*p* = 0.002) or Modic type (*p* = 0.025).

**Conclusions:**

Improvement of LBP on the affected side while the patient is in motion suggests that radicular LBP is improved following discectomy by nerve root decompression. Furthermore, residual LBP may reflect increased load and pressure on the disc and endplate in the sitting position.

## Background

Lumbar disc herniation (LDH) is one of the most common causes of low back pain (LBP) and sciatica. Surgical treatment is well known to be beneficial for patients with LDH who fail to respond to conservative care. Favorable results for LBP, lower extremity pain (LEP), and lower extremity numbness (LEN) in patients with LDH treated with discectomy have been demonstrated [1–4]. In most of those studies, only a conventional visual analog scale (VAS) and the Oswestry disability index (ODI) were used to determine LBP. Those studies were unable to determine detailed changes in LBP and the characteristics of relief and residual LBP. By contrast, recent reports have clarified the characteristics of LBP using a detailed VAS for some lumbar degenerative disorders, including spondylolysis and lumbar spinal stenosis (LSS) [5–7]. Nevertheless, there are no reports of the detailed changes and location of LBP before and after discectomy for LDH. Thus, we conducted an observational study to clarify the detailed characteristics and location of LBP, LEP, and LEN before and after discectomy for LDH using detailed VAS bilaterally.

## Methods

### Patient selection

The present study was approved by the ethics committee of Toho University Sakura Medical Center (No. 2012071). Informed consent was obtained from all patients. In the present study, we enrolled a total of 114 patients under the age of 75 who were treated with discectomy for LDH from April 2010 to March 2018. LDH was diagnosed by 4 orthopedic spine surgeons (HT, YaA, MI, JS) based on neurological findings, the presence of persistent and unremitting LBP for more than 3 months, X-ray images, and magnetic resonance imaging (MRI). All the patients were treated conservatively. Those who were not improved by sufficient conservative treatment and wished to undergo surgical treatment were included in this study. Patients with LSS or degenerative spondylolisthesis (DS) comorbidities were excluded. Patients who were diagnosed with a lateral herniation treated with fusion surgery were excluded, as were those with thoracic myelopathy and hip osteoarthritis. Patients with recurrent herniation were excluded, and unfortunately so were those who had a recurrence of herniation and underwent revision surgery within 1 year of their primary surgery. The categories of patients excluded from the study due to these complications, are broken down as follows: 5 patients with LSS or DS, 7 patients with lateral herniation, 1 patient with thoracic myelopathy, 1 patient with hip osteoarthritis, and 7 patients with recurrent herniation. Unfortunately, 5 patients (4.3%) had a rapid (less than one -year post op) recurrence of herniation. In addition, 5 patients were excluded because of a lack of data and 18 patients because of a loss to follow-up. Ultimately, data from 65 patients were included in the present study.

### Discectomy procedure

All the surgical procedures were performed under an operating microscope as described in detail elsewhere [8]. We performed 59 of 65 surgeries using a hemi approach. In the first 31 patients, the surgeries were performed using the conventional microdiscectomy. In the 28 patients treated since 2015, the surgeries were performed using a tubular retractor (METRx MD system, Medtronic, US) to minimize damage to the paraspinal muscles on the approached side. Six of the patients experienced bilateral symptoms with a central herniation. Therefore, we performed bilateral fenestration and bilateral extirpation of herniation.

### Clinical outcome

**Fig. 1:**
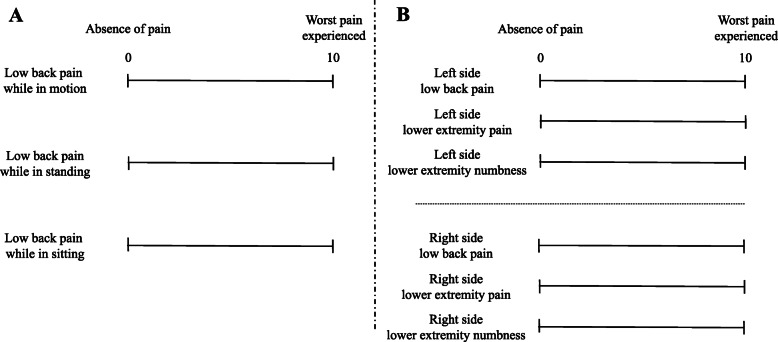
VAS scores. **a** Detailed LBP VAS (0–100 mm) scores. LBP was scored independently under 3 different postural conditions: in-motion, standing, and sitting. **b** LBP, LEP, and LEN VAS (0–100 mm) scores bilaterally on the approached and opposite sides

In this study, buttock pain was included in LEP and, therefore, the definition of LBP in this study does not include buttock pain. Based on past reports, a detailed VAS (100 mm) score for LBP was obtained under 3 conditions: while the patient was in motion, standing, and sitting (Fig. 1a) [5, 6]. In addition, the location (left versus right side) of LBP, LEP, and LEN were determined and analysis performed of the affected (approached) and opposite side (Fig. 1b) [6]. At the time of the outpatient examination, we carefully explained how to complete the questionnaire to each patient, especially for the LBP in the 3 different situations (in motion, while standing, and while sitting) and the point that LBP did not include buttock pain. In the patients with bilateral symptoms who underwent bilateral laminectomy and herniation extirpation, we established the affected side as the one that was more symptomatic and more extensively herniated on MRI. The ODI was also used to assess clinical improvement and included activities of daily living. All VAS and ODI values were determined before surgery, at 3 months, at 6 months, and at 1-year of follow-up. We investigated the relationship between the VAS scores and surgical level as well as in the following 3 surgical groups: conventional discectomy with conventional hemi-laminectomy (C), discectomy using a tubular retractor (T), and discectomy with bilateral laminectomy (B).

### Imaging

We analyzed MRI before surgery and at the 1-year follow-up, except for 3 patients who were unable to undergo MRI because of a pacemaker insertion (1 patient) or claustrophobia (2 patients). In total, we analyzed MRI from 62 patients. We determined disc degeneration using the Pfirrmann grading [9]. Vertebral endplate changes were determined using Modic type [10]. All the MRI analyses were conducted inependently by 3 examiners (YE, KI, and KoM) who were blinded to clinical data from the patients. The patients were divided into 2 groups: C group and N group according to their changes of Pfirrmann grade or Modic type before and after discectomy. Differences between the 2 groups were determined.

### Statistical analyses

Results are presented as the mean (standard deviation). A paired *t* test was used to compare each detailed VAS score before and after surgery. A one-factor ANOVA was used to compare the relationship between VAS scores and the 3 different surgical levels and procedures. A repeated measures ANOVA followed by a post hoc Turkey–Kramer test was used to determine changes over time for each VAS and the ODI. A Student *t* test was used to compare changes in Pfirrmann grade or Modic type with residual LBP, as measured by VAS. We considered *p* < 0.05 significant in the tests of statistical inference. All statistical analyses were performed using JMP software (version. 14.2.0, SAS Institute, Cary, NC, USA).

## Results

### Patient characteristics

Table 1 shows the characteristics of the 65 patients whose data was included in the present study. The ratio of males to females was 1.097. Herniations at L4–5 and L5-S were more frequent than at L3–4 in this series. There were no cases of L1–2 or L2–3 herniation included in this study. Disc degeneration by Pfirrmann grading showed that grade 2 was most frequent and there were no cases of grade 1. Fourteen patients showed endplate changes as determined by Modic type. There were no cases of surgical site infection in this series, nor were there other critical complications such as thromboembolic events or nerve root injuries.

**Table 1:** Patient characteristecs

Age	43.6 (14.7)
Sex	
Male	34
Female	31
Dominant side	
Lt	33
Rt	26
Blt	6
Herniation level	
L3/4	6
L4/5	28
L5/S	31
Pfirrmann classification before surgery	
2	2
3	39
4	19
5	2
Unable	3
Modic type before surgery	
None	48
1	3
2	10
3	1
Unable	3
Surgidal procedure	
C	31
T	28
B	6

C: discectomy with conventional hemi laminectomy

T: discectomy using a tubular retractor

B: discectomy with bilateral laminectomy

### Clinical outcomes (VAS and ODI)

The time course changes of detailed LBP VAS scores are shown in Table 2. Before surgery, LBP while the patient was in motion was significantly higher than LBP while they were sitting (paired *t* test, t(64) = 1.997, *p* = 0.025). The increased LBP found while they were in motion as well as the LBP while they were standing and sitting was significantly improved following discectomy (repeated majors ANOVA, *F*(3, 2) = 315.5, *p* < 0.001). By contrast, at 1 year after surgery, the residual LBP while they were sitting was significantly higher than the LBP while they were in motion or standing (paired *t* test, t(64) = 2.200, *p* = 0.015). The time course changes of bilateral LBP, LEP, and LEN VAS scores were shown in Table 3. LBP on the affected side was significantly higher than that on the opposite side (paired *t* test, t(64) = 6.848, *p* < 0.001). The LBP on the affected side was improved significantly following surgery, and LBP relief was maintained on both sides until the 1 year follow-up (repeated measures ANOVA, *F*(3, 1) = 44.29, *p* < 0.001). LEP and LEN on the affected side were also significantly greater than before surgery (paired *t*-test, LEP: t(64) = 14.32, LEN: t(64) = 10.28, *p* < 0.001). Significant improvements of LEP and LEN on the affected side were shown and the relief was maintained on both sides until the 1 year follow-up (repeated measures ANOVA, LEP: *F*(3, 1) = 174.5, LEN: *F*(3, 1) = 107.2, *p* < 0.001), although mild LEP and LEN remained on the affected side. ODI also showed significant improvements following discectomy (Table 4).

**Table 2:** Time course changes of detailed LBP VAS scores

LBP	Before surgery	3 months after surgery	6 months after surgery	1 year after surgery
in motion	62.8 (29.9)*	15.5 (20.1)	12.5 (18.2)	13.2 (19.5)
while standing	61.6 (31.6)	12.7 (16.7)	11.9 (16.5)	13.8 (18.9)
while sitting	56.6 (32.9)	14.8 (19.2)	13.8 (18.5)	17.3 (22.0)†

Reported as mean (SD)

* Significantly higher than LBP in sitting (paired t-test, t(64) = 1.997, *p* = 0.025)

† Significantly higher than LBP in in motion (paired t-test, t(64) = 2.200, *p* = 0.015)

**Table 3:** Time course changes of bilateral VAS

	Before surgery	3 months after surgery	6 months after surgery	1 year after surgery
LBP				
on affected side	46.9 (34.0)*	11.2 (16.0)	12.3 (19.0)	13.8 (20.1)
on opposite side	17.4 (27.0)	7.20 (13.4)	9.22 (18.3)	12.7 (20.6)
LEP				
on affected side	77.5 (25.2)**	16.6 (21.6)	10.0 (15.4)	8.92 (15.2)
on opposite side	12.0 (27.5)	5.13 (13.3)	4.53 (11.6)	5.77 (13.0)
LEN				
on affected side	62.7 (32.7)†	13.4 (19.4)	10.7 (15.7)	11.4 (17.6)
on opposite side	12.3 (28.0)	3.63 (11.6)	3.36 (8.73)	2.74 (12.4)

Reported as mean (SD)

* Significantly higher than LBP on opposite side (t(64) = 6.848, *p* < 0.001)

** Significantly higher than LEP on opposite side (t(64) = 14.32, *p* < 0.001)

† Significantly higher than LEN on opposite side (t(64) = 10.28, *p* < 0.001)

**Table 4:** Time course changes of ODI

	Before surgery	3 months after surgery	6 months after surgery	1 year after surgery
ODI	51.2 (20.2)	14.3 (12.6)	12.3 (19.0)	13.8 (20.1)*

Reported as mean (SD)

* Significantly improved by surgery (t(64) = 14.06, *p* < 0.001)

**Fig. 2:**
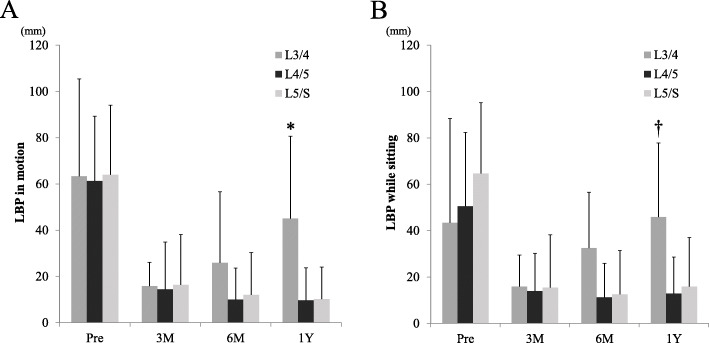
**a** Changes in LBP during motion and surgical levels. LBP during motion at 1 year after surgery was significantly greater in cases of herniation at the L3/4 level (*one factor ANOVA, *F* = 11.87, *p* < 0.001). **b** Changes in LBP while sitting and surgical levels. LBP while sitting at 1 year after surgery was also significantly greater in cases of L3/4 herniation level (†one factor ANOVA, *F* = 6.735, *p* = 0.002)

Taking the results of the detailed LBP VAS scores into account, we investigated the temporal changes in LBP VAS scores while the patient was in motion and while they were sitting. The time course of changes of the detailed LBP VAS and surgical level are shown in Fig. 2. There was no significant difference in LBP before surgery at the various surgical levels. However, the residual LBP VAS score while the patient was in motion and while they were sitting was significantly increased at the L3–4 level at 1 year after surgery (one factor ANOVA, motion: *F* = 11.87, *p* < 0.001, sitting: *F* = 6.735, *p* = 0.002).

**Fig. 3:**
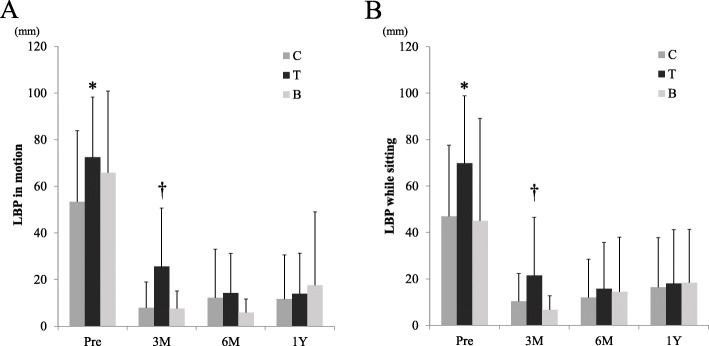
**a** Changes in LBP during motion and surgical procedures. **b** Changes in LBP while sitting and surgical procedures. C: conventional discectomy, T: microscopic discectomy using a tubular retractor, B: bilateral laminectomy and discectomy. LBP both during motion and while sitting before surgery were significantly greater in group T (*one factor ANOVA, *F* = 3.246, *p* = 0.046), and the residual LBP at 3 months after surgery was significantly greater in group T (†one factor ANOVA, *F* = 7.519, *p* = 0.001). However, LBP was improved with all 3 surgical procedures and LBP became equally level at 1 year after surgery (one factor ANOVA, *F* = 0.263, *p* = 0.770)

Time course changes in detailed LBP VAS scores and surgical procedures (C, T, and B groups) were shown in Fig. 3. LBP while the patients were in motion was significantly higher before surgery in those of group T (one factor ANOVA, *F* = 3.246, *p* = 0.046), and therefore the residual LBP at 3 months after surgery was also higher in those in group T (one factor ANOVA, *F* = 7.519, *p* = 0.001). However, at 1 year after discectomy, the residual LBP became almost equal for all 3 surgical procedures (one factor ANOVA, *F* = 0.263, *p* = 0.770).

### Correlation between MRI findings and VAS

The time course of changes in Pfirrmann grade and Modic type are shown in Table 5. Overall, significant changes in both Pfirrmann grade and Modic type were observed (Chi-squared test, Pfirrmann: X2 (6, *N* = 62) = 55.46, Modic: X2 (9, *N* = 62) = 50.61, *p* < 0.001). Seventeen patients had changes in Pfirrmann grade following discectomy. We divided them into 2 groups: Pfirrmann grade changing (PC group) and not changing (PN group). After discectomy, Modic type changed in 12 patients, and we also divided them into 2 groups: Modic type changing (MC group) and not changing (MN group).

**Table 5:** Time course changes of

A. Pfirrmann grade						
		1 year after surgery				
		2	3	4	5	Total
Before surgery	2		2			2
	3		26	13		39
	4			17	2	19
	5				2	2
	Total		28	30	4	62
B. Modic type						
		1 year after surgery				
		None	1	2	3	Total
Before surgery	None	40	4	3	1	48
	1		2	1		3
	2		1	8	1	10
	3		1			1
	Total	40	8	12	2	62

**Fig. 4:**
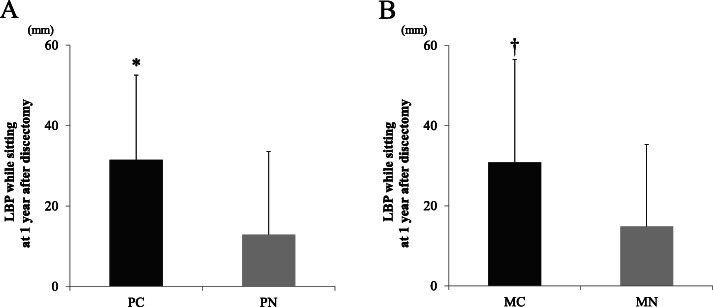
**a** Relationship between LBP while sitting at 1 year after surgery and Pfirrmann classification changes. PC: Pfirrmann classification changing group, PN: Pfirrmann not changing classification group. Residual LBP while sitting in the PC group was significantly greater than that in the PN group (*student t test, t(60) = 3.138, *p* = 0.002). **b** Relationship between LBP while sitting at 1 year after surgery and Modic type changes. MC: Modic type changing group, MN: Modic type not changing group. The residual LBP while sitting in the MC group was significantly greater than that in the MN group (†student t test, t(60) = 2.304, *p* = 0.025)

Considering that residual LBP while the patient was sitting was increased at 1 year after discectomy, we investigated the relationship between the Pfirrmann grade, Modic type, and LBP VAS score while the patients were sitting at 1 year after discectomy. LBP VAS scores while they were sitting at 1 year after discectomy were 31.5 (21.1) for those in the PC group and 12.9 (20.7) for those in the PN group; notably, they were significantly higher in those in the PC group (t(60) = 3.138, *p* = 0.002, Fig. 4a). In addition, LBP VAS scores while they were sitting at 1 year after discectomy were 30.8 (25.7) for those in the MC group and 14.9 (20.5) for those in the MN group, showing the scores were significantly higher in those in the MC group (t(60) = 2.304, *p* = 0.025, Fig. 4b).

## Discussion

To our knowledge, this study is the first to determine the characteristics and location of LBP using a detailed VAS we developed in patients with LDH treated with discectomy who did not experience a recurrence. According to a previous study that analyzed detailed and bilateral VAS scores for LSS patients, LBP in patients with LSS before surgery were significantly greater while the patient was standing, but pain was reduced by decompression surgery, with LBP improving equally on the affected and opposite sides [6]. The first noteworthy point of the present study is that LBP while the patients were in motion was significantly greater in those with LDH before surgery, and the LBP while they were in motion on the affected side was reduced by discectomy. This pattern of LBP relief suggests that radicular LBP is improved by nerve root decompression surgery, as indicated in previous reports [1, 6]. However, despite this similarity regarding nerve root decompression, the greater LBP that occurred in patients with LDH while they were in motion was distinct from the increased LBP found while patients with LSS were standing. For this reason, we speculate that nerve root compression in patients with LDH usually occurs with a more acute onset than that in patients with LSS. In addition, this difference in LBP characteristics may be influenced by the degree of disc and endplate degeneration in patients with LDH compared with those with LSS because patients with LDH tend to be younger than those with LSS.

Results from the present study also suggest that residual LBP after discectomy in patients with LDH who did not experience a recurrence was most pronounced while the patient was sitting. A recent report indicated that higher intradiscal pressure while sitting may result in LBP in the presence of lumbar degenerative disc diseases [11]. Pathological mechanisms of discogenic low back pain included sensory nerve ingrowth into the disc, upregulation of neurotrophic factors like nerve growth factor and inflammatory cytokines, and mechanical stress [12, 13]. Our findings of residual LBP while the patients were sitting and changes in Pfirrmann grade, when taken in combination, may suggest that the residual LBP was associated with increased load and pressure on the disc in the sitting position. Alternatively, it is also well known that Modic changes influence LBP [14]. Ohtori et al. reported favorable surgical outcomes for LDH complicated with Modic type I [15]. Although LEP improvement was obtained in patients with Modic change in our study, the residual LBP in the MC group leads us to believe that changes in load and inflammation at the endplate may also cause residual LBP while the patient is sitting.

Recent reports indicated that performing a minimally invasive discectomy using a tubular retractor under a microscope or endoscope is feasible for the treatment of LDH [16, 17]. In the present study, we compared these 3 surgical procedures, including conventional discectomy. Residual LBP at 3 months after surgery was greater in patients in group T because the baseline of LBP before surgery was significantly greater in those in this group. However, the residual LBP at 1 year follow up was equal following all 3 surgical procedures. This, along with previous reports, suggests that surgical invasion of the paraspinal muscles does not influence residual LBP [6, 16]. In addition, in the analysis of the residual LBP at 1 year follow up except for cases of bilateral laminectomy (group B), we found similarly that LBP while the patient is sitting was significantly higher than the LBP found when the patient was in motion or standing. Furthermore, recent reports indicated that the surgical procedure (open discectomy versus micro discectomy) did not influence the surgical outcome for the residual pain [18, 19]. Although including the 3 different surgical methods may present the bias in the present study, we speculate that the type of surgical procedures has less of an impact on the result for the residual LBP.

While no reports describing the relationship between surgical levels and residual LBP were found, in the present study, residual LBP was significantly greater in patients with herniations at L3–4. It is difficult to explain this phenomenon. However, we speculate that this may have been because patients with L3–4 herniations were highly complicated and also had L4–5 or L5-S disc degenerations. Further investigation with a larger sample size including multivariate modeling or mediation analysis is needed to understand this residual LBP.

The present study has several limitations. First, the present study is observational, and we did not evaluate detailed and bilateral LBP VAS scores of patients who underwent conservative treatment alone. In our study some of the patients underwent conservative treatment at another hospital, and they wished to undergo surgical treatment as soon as possible, leaving no time to evaluate further conservative treatment. Further prospective investigation will be needed to clarify this point. Second, in the present study, we investigated only ODI as a patient-based outcome and did not investigate other patient-based outcomes such as Short Form 36 (SF-36), EuroQol 5 dimension (EQ-5D), and Japanese Orthopaedic Association back pain evaluation questionnaire (JOA BPEQ). Although such patient-based outcomes as SF-36, EQ-5D, and JOA BPEQ are also important, the main purpose of this study was to determine the degree of detailed LBP under various conditions (while the patient was in motion, sitting, and standing). However, patient-based outcomes could not determine the LBP in various conditions and may include postsurgical psychogenic factors. Furthermore, excessive quantities of questionnaire may place the extra stress on the patients. To simplify the result and avoid the excessive stress on the patients, we only investigated VAS scores and ODI. Third, the present study excluded patients complicated with dynamic instability or patients with lateral herniations who underwent fusion surgery because we wanted to avoid LBP caused by instability of discs and facet joints [20]. Furthermore, the present study also excluded patients who, unfortunately, had a recurrence of herniation in the short term (less than a year postoperatively) because we wanted to determine residual LBP in the absence of herniation recurrence. If those cases had been included, the results would have been confounded because they had high VAS scores under all 3 conditions. Fourth, the present study did not evaluate sagittal alignment. Sagittal imbalance such as pelvic incidence and lumbar lordosis mismatch may contribute to postoperative LBP [21]. Using detailed VAS scores, Aoki et al. indicated that sagittal imbalance after a short segment fusion surgery resulted in residual LBP while the patient was standing [22]. Considering our finding that residual LBP while the patient was sitting was present at 1 year after discectomy, we speculate that residual LBP is less affected by sagittal alignment. Finally, the follow up after discectomy was incomplete. When patients undergo discectomy and have significant pain relief, they sometimes drop out of care at the outpatient clinic. In the present study, 18 of 114 (15.7%) patient participants dropped out. Generally, 2 years of follow-up is recommended for this type of study. However, in the present study, we were compelled to set the follow-up period to 1 year because of a decreasing follow-up rate. Further investigation, such as a prospective cohort study that follows all the cases fully will be needed to resolve this follow-up issue.

## Conclusions

In patients with LDH without recurrence, LBP on the affected side while they are in motion was significantly greater before surgery and was reduced following discectomy. This LBP relief suggests a radicular nature to the LBP that is improved by nerve root decompression surgery. The residual LBP at 1 year after discectomy was most dominant while they were sitting. This, in combination with our findings regarding the relationship between residual LBP and changes in Pfirrmann grade and Modic type, may suggest that the residual LBP was associated with increased load and pressure on the disc in the sitting position.

## Data Availability

The datasets used during the current study are available from the corresponding author on reasonable request.

## Abbreviations

LDH: Lumbar disc herniation
LBP: Low back pain
LEP: Lower extremity pain
LEN: Lower extremity numbness
VAS: Visual analog scale
ODI: Oswestry disability index
LSS: Lumbar spinal stenosis
MRI: Magnetic resonance imaging
DS: Degenerative spondylolisthesis
SF-36: Short Form 36
EQ-5D: EuroQOL 5 dimension
JOA BPEQ: Japanese Orthopaedic Association back pain evaluation questionnaire
